# Organellar quality control crosstalk in aging‐related disease: Innovation to pave the way

**DOI:** 10.1111/acel.14447

**Published:** 2024-12-12

**Authors:** Yu Li, Jinxin Qi, Linhong Guo, Xian Jiang, Gu He

**Affiliations:** ^1^ Department of Dermatology & Venerology West China Hospital, Sichuan University Chengdu China; ^2^ Laboratory of Dermatology, Clinical Institute of Inflammation and Immunology, Frontiers Science Center for Disease‐Related Molecular Network, State Key Laboratory of Biotherapy West China Hospital, Sichuan University Chengdu China

**Keywords:** aging‐related diseases, cellular organelle, crosstalk, quality control

## Abstract

Organellar homeostasis and crosstalks within a cell have emerged as essential regulatory and determining factors for the survival and functions of cells. In response to various stimuli, cells can activate the organellar quality control systems (QCS) to maintain homeostasis. Numerous studies have demonstrated that dysfunction of QCS can lead to various aging‐related diseases such as neurodegenerative, pulmonary, cardiometabolic diseases and cancers. However, the interplay between QCS and their potential role in these diseases are poorly understood. In this review, we present an overview of the current findings of QCS and their crosstalk, encompassing mitochondria, endoplasmic reticulum, Golgi apparatus, ribosomes, peroxisomes, lipid droplets, and lysosomes as well as the aberrant interplays among these organelles that contributes to the onset and progression of aging‐related disorders. Furthermore, potential therapeutic approaches based on these quality control interactions are discussed. Our perspectives can enhance insights into the regulatory networks underlying QCS and the pathology of aging and aging‐related diseases, which may pave the way for the development of novel therapeutic targets.

## Graphical abstract

Organellar homeostasis and inter‐organelle crosstalk are vital for cell survival. Quality control systems (QCS) maintain this balance, and their dysfunction is linked to aging‐related diseases like neurodegeneration and cancer. Our review explores QCS interactions among organelles and discusses potential therapies, aiming to identify new therapeutic targets.

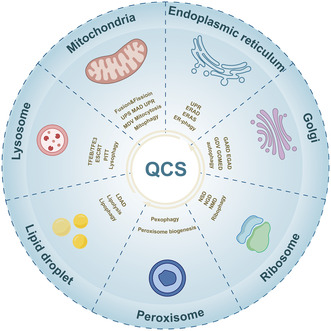

## 1 INTRODUCTION

**FIGURE 1:**
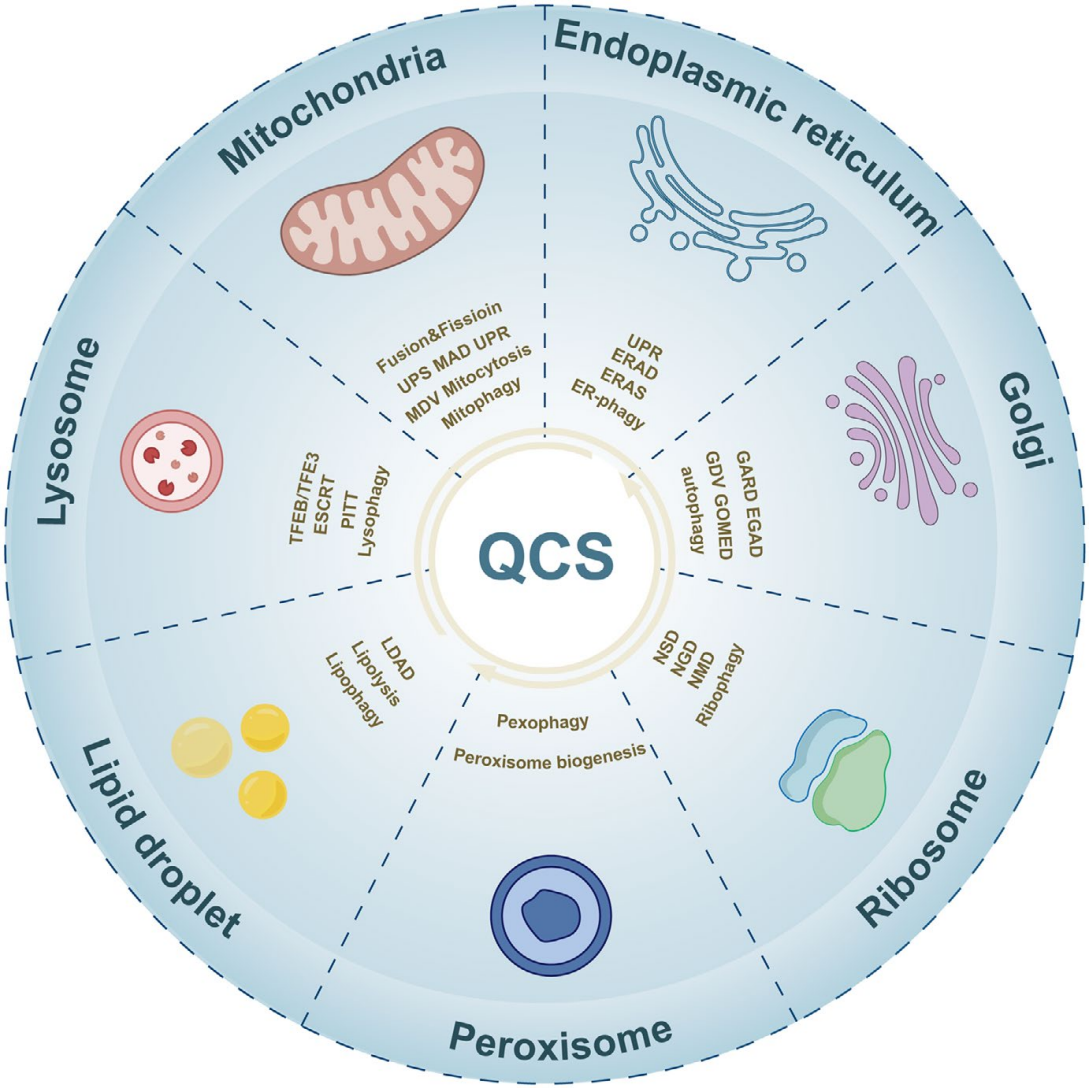
The overview of organellar quality control systems. EGAD, endosome and Golgi‐associated degradation; ERAD, ER‐associated degradation; ERAS, ER‐associated RNA silencing; ESCRT, endosomal sorting complex required for transport; GARD, Golgi apparatus‐related degradation; GDV, Golgi‐derived vesicle; GOMED, Golgi membrane‐associated degradation; LDAD, LD‐associated degradation; MAD, mitochondrial‐associated degradation; MDV, mitochondrial‐derived vesicle; NGD, no‐go decay; NMD, nonsense‐mediated decay; NSD, non‐stop decay; PITT, phosphoinositide‐initiated membrane tethering and lipid transport; TFEB, transcription factor EB; UPR, unfolded protein response; UPS, ubiquitin‐proteasome system.

Organelles, which consist of intracellular organelles and extracellular organelles, are tiny structures that perform specific physiological functions in cells (Huang et al., 2022). The former contain the nucleus, mitochondria, endoplasmic reticulum (ER), ribosome, Golgi, peroxisome, lysosome, autophagosome, and lipid droplet (LD), while the extracellular organelles are primarily extracellular vesicles (EVs) secreted by living cells (Harapas et al., 2022; Hu et al., 2023). Overstressed or damaged cellular organelles have been reported to participate in biological activity and even determine the fate of cells, and the disruption of organelles is crucial in the pathogenesis and progression of many aging‐related diseases, such as neurodegenerative diseases, cardiovascular diseases, and cancers (Chan, 2020). Therefore, to maintain and restore homeostasis, cells have evolved multiple quality control systems (QCS), including mitochondrial, ER, Golgi, peroxisome, LD, and lysosome‐derived quality control (Figure 1).

In fact, organelles can interact to sustain cellular homeostasis through mechanisms such as membrane contact sites (MCSs) or vesicular transport, facilitating the exchange of necessary contents for normal cellular functions (Phillips & Voeltz, 2016; Shanmughapriya et al., 2020). For example, the mitochondria‐associated membrane (MAM), also known as the mitochondria‐ER contact site (MERCS), is a junction (<50 μm) connecting mitochondria and the ER, representing a platform for thousands of proteins regulating multiple responses, including mitochondrial quality control (MQC), calcium ion homeostasis, lipid transfer, and oxidative stress (Missiroli et al., 2018). Recent novel technologies, such as precisely isolating populations and components of cells and high‐resolution imaging techniques, have enabled researchers to deepen the understanding of how organelles within individual cells and tissues respond and adapt to health and disease (Harbauer et al., 2022; Zhang, Yu, et al., 2023). Increasing evidence indicates that an imbalance in organelle interactions can lead to various pathological conditions (Wong et al., 2019). Using high‐resolution microscopy and proteomic analysis, dysfunction of the structure and protein landscape of MAMs has been found to be involved in the pathogenesis of aging and aging‐related diseases (Janikiewicz et al., 2018; Missiroli et al., 2018). The detailed effects and mechanisms of MAMs in quality control will be discussed later.

Given the complexity of organelle crosstalk and the lack of comprehensive investigations, this review focuses on QCS and the crosstalk between intracellular organelles that occur during normal physiological processes and disease progression and also discuss potential therapeutic targets for QCS in various aging‐related diseases.

## 2 QUALITY CONTROL OF INTRACELLULAR ORGANELLES

### 2.1 Mitochondrial quality control

Mitochondria are the cellular hubs for energy metabolism and many essential cellular processes. Among the double‐membrane components, 13 proteins are encoded by mitochondrial DNA (mtDNA), while approximately 1500 additional mitochondrial proteins orchestrate normal mitochondrial morphology and functions (Al Amir Dache & Thierry, 2023; Ng et al., 2021). Notably, as the cornerstone of energy metabolism, reactive oxygen species (ROS) are natural byproducts that can induce oxidative stress to disrupt cellular structures, including mtDNA (Roger et al., 2017; Sreedhar et al., 2020). To mitigate mitochondrial damage, various MQC systems, including mitochondrial fusion and fission, mitochondrial‐associated degradation (MAD), the ubiquitin (Ub)‐proteasome system (UPS), mitochondrial chaperones and proteases, unfolded protein response (UPR^mt^), mitochondrial‐derived vesicles (MDVs), mitocytosis, and mitophagy, have evolved. These safeguard mechanisms overlap or interact to maintain mitochondrial homeostasis to ensure that mitochondria function correctly, as described in previous studies (Ni et al., 2015; Ashrafi & Schwarz, 2013; Ng et al., 2021; Song et al., 2021).

In brief, MAD and the UPS act as quality control checkpoints for importing and folding mitochondrial proteins on the outer mitochondrial membrane (OMM) and some nonimported proteins. Once inside the mitochondria, chaperones and proteases manage the quality of imported proteins. Finally, the UPR^mt^, MDVs, and mitophagy monitor and remove mitochondrial fragments or damaged mitochondria in response to diverse cellular signals (Ng et al., 2021; Song et al., 2021).

In addition, emerging evidence suggests that damaged mitochondria can generate MDVs that transport cargo to migrasomes, which are then released from mammalian cells through EVs. This indicates that the selective release of dysfunctional mitochondria, namely, mitocytosis, is also a QCS for mitochondria (Jiao et al., 2021; Pan et al., 2023).

### 2.2 ER quality control

The ER is an extensive and vigorous structure essential for protein synthesis, calcium balance, and lipid metabolism. As a continuous membrane compartment, the shape and distribution of ER are controlled by numerous integral membrane proteins, as well as interactions with other organelles and the cytoskeleton (Schwarz & Blower, 2016).

The ER manages proteostasis, including biogenesis, folding, transport, and degradation of most proteins in cells. However, numerous genetic and environmental factors disturb the normal functions of the ER, resulting in the aggregation of misfolded proteins and consequent ER stress (ERS) (Oakes & Papa, 2015). To maintain ER homeostasis and regulate cell fate and function, cells have evolved protein QCS through different pathways, including the unfolded protein response (UPR), ER‐associated degradation (ERAD), and ER‐phagy pathways (Hwang & Qi, 2018; Wang & Kaufman, 2016).

Previous studies have reviewed the above ER quality control (ERQC) pathways (Wang & Kaufman, 2016). In brief, the UPR^ER^ consists of three signaling pathways, including double‐stranded RNA‐activated protein kinase (PKR)‐like ER kinase (PERK), activating transcription factor 6 (ATF6), and inositol‐requiring enzyme 1 (IRE1α), which detect ERS and activate downstream pathways to assist in ER homeostasis and protein folding (Wiseman et al., 2022). In addition, aberrant proteins are moved back into the cytoplasm via canonical ERAD and then fragmented by UPS as well as degraded by UPR transducer‐induced autophagy (noncanonical ERAD pathway). Nevertheless, not all abnormal proteins can be eliminated by these pathways. Under such circumstances, ER‐phagy is activated (Chino & Mizushima, 2020; Ferro‐Novick et al., 2021; Hetz & Papa, 2018). Additionally, UPR transducers can activate ER‐phagy while ERAD can adjust the UPR in turn by controlling protein turnover and the abundance of IRE1α to coordinate the crosstalk of different QCS (Hwang & Qi, 2018; Senft & Ronai, 2015; Song et al., 2017). Notably, UFMylation, a novel ubiquitin‐like posttranslational modification (PTM) dependent on the ubiquitin‐fold modifier 1 (UFM1), links various ERQC. Recent studies have revealed that ER‐localized UFMylation could inhibit the IRE1α‐mediated UPR^ER^ and ERAD during ER‐phagy (Cherubini & Zito, 2022; Ishimura et al., 2023; Makhlouf et al., 2024). This underscores the intricate interplay between ERS, UPR, and the additional ERQC pathways.

A recent study also identified a novel mechanism of ER‐associated RNA silencing (ERAS) by the Argonaute protein (AGO) RNAi‐deficient‐1 (RDE‐1)/AGO2, which coordinates with ERAD to preserve ER homeostasis (Efstathiou et al., 2022).

### 2.3 Golgi quality control

The Golgi apparatus is composed of multiple cisternae. Typically, the Golgi acts as a station to receive proteins and lipids from the ER, facilitates PTM, and then delivers to other cell compartments. In instances where the Golgi fails to meet the cellular demands adequately, it triggers the Golgi stress response. Similar to other organelles, Golgi quality control is important for maintaining homeostasis, but the exact underlying mechanisms still need to be further explored (Chang & Yang, 2022; Gao et al., 2021).

Studies have uncovered that the Golgi serves to target misfolded proteins for degradation through Golgi apparatus‐related degradation (GARD), endosome and Golgi‐associated degradation (EGAD), Golgi‐derived vesicles (GDVs), Golgi membrane‐associated degradation (GOMED), and selective autophagy of the Golgi (Chang & Yang, 2022; Schwabl & Teis, 2022). These mechanisms of Golgi quality control are similar to those of the ER in which misfolded proteins are retrogradely transported from the Golgi to the ER. These proteins are either degraded via the proteasome or directed to lysosomes for degradation, thereby preserving cellular protein homeostasis.

### 2.4 Ribosome quality control

Ribosomes, as dynamic macromolecular complexes, are the main cellular apparatus for protein synthesis. In mammals, they are present at more than ten million copies per cell and are structurally heterogeneous (Emmott et al., 2019; Genuth & Barna, 2018). However, once aberrant proteins or messenger RNAs (mRNAs) continuously accumulate, they can have negative effects on cell fate and death. In addition, upon exposure to stimuli, ribosomes may malfunction and cause abnormal protein or mRNA accumulation, which leads to a series of diseases called ribosomopathies (Joazeiro, 2019).

Correspondingly, to monitor protein synthesis and function ribosomes, the ribosome quality control (RQC) has evolved through the entire process from ribosome biogenesis to degradation. In general, RQC degrades nascent peptides via 26S proteasome and error mRNAs by three surveillance mechanisms including non‐stop decay (NSD), no‐go decay (NGD), and nonsense‐mediated decay (NMD), while the E3 ubiquitin ligase listerin (Ltn1) plays a key role in RQC (Alagar Boopathy et al., 2023; Zhao et al., 2022).

The induction of ribophagy has been demonstrated through pharmacological inhibition of mTOR1, nutrient starvation, and arsenite. Nuclear FMR1interacting protein 1 (NUFIP1), a mammalian ribophagy receptor, directly engages with LC3B and ribosomes to promote ribophagy. Surprisingly, recent findings demonstrate that the absence of NUFIP1 didn't hinder ribophagy. Moreover, under conditions of starvation and mTOR inhibition, ribophagy appears to have minimal effect on ribosomal abundance, suggesting a limited influence of ribophagy on overall ribosome function and protein synthesis. Thus, further research is warranted to elucidate the constituents and functions of mammalian ribophagy (Deng et al., 2017; Denton & Kumar, 2018; Vargas et al., 2023).

### 2.5 Peroxisome quality control

Peroxisomes can arise via multiple pathways, including de novo genesis from the ER, fusion between vesicles originating from mitochondria and ER, or through the proliferation and division of existing organelles (Di Cara et al., 2023). The regulation of peroxisome abundance involves intricate processes such as peroxisome biogenesis and degradation (pexophagy). More than 30 peroxin (PEX) proteins are essential for maintaining peroxisomal size and function within cells (Farré et al., 2019). Despite this, the absence of a specific pexophagy receptor in mammalian cells suggests that mammalian pexophagy primarily relies on the ubiquitination of peroxisome‐associated proteins and subsequent interactions with autophagic receptors, including SQSTM1/p62 and NBR1 (Jo, Park, & Cho, 2020).

### 2.6 Lipid droplet quality control

LDs serve as central sites for lipid metabolism derived from ER (Bersuker & Olzmann, 2017). LDs establish physical connections with other organelles through MCS, facilitating the transfer of free fatty acids (FFAs) for β‐oxidation (Herker et al., 2021). Current research suggests that LDs can be selectively degraded by lipolysis or lipophagy via macrolipophagy and microlipophagy pathways, especially in the liver. The former one is dependent of ATP and Rab proteins driven by cellular energetic stress, whereas the latter primarily relies on ATP induced by nitrogen starvation or glucose depletion (Filali‐Mouncef et al., 2022; Zheng, 2018). Furthermore, the involvement of ubiquitination with LDs suggests the existence of an LD‐associated degradation (LDAD) pathway, as previously reviewed (Roberts & Olzmann, 2020).

### 2.7 Lysosome quality control

Lysosomes are known as terminal degradation stations for degrading toxic or unwanted substances in cells. In addition, they cooperate extensively to mediate fundamental cellular activities such as signal transduction, plasma membrane repair, metabolic adaptation, and immunity (Perera & Zoncu, 2016; Settembre et al., 2013).

It is now clear that once lysosomal membrane permeabilization (LMP) is disrupted, the integrity of lysosomes is compromised, leading to detrimental outcomes such as lysosomal leakage and cell death (Lawrence & Zoncu, 2019). The occurrence of lysosomal leakage initiates numerous lysosomal QCS, including lysophagy, the transcription factor EB (TFEB)/TFE3 pathway, the endosomal sorting complex required for transport (ESCRT) pathway, and the phosphoinositide‐initiated membrane tethering and lipid transport (PITT) pathway (H. Yang & Tan, 2023; Sardana & Emr, 2021).

## 3 CROSSTALK OF QUALITY CONTROL BETWEEN MULTIPLE ORGANELLES

Cellular homeostasis relies on the collaborative interaction and communication among organelles, facilitating swift exchange of materials and information and orchestrating diverse biological processes across varying environmental conditions. The integrity of this organelle crosstalk network is paramount for maintaining the internal milieu while the disruptions of organelle communication may result in multiple pathological processes (Bravo‐Sagua et al., 2014; Inoue et al., 2019). Nonetheless, the mechanisms and functions of these interactions remain poorly understood. Here, we review and discuss the crosstalk between different organellar QCS to elucidate the complete network of inter‐organellar communications.

### 3.1 Crosstalk between mitochondria and the ER

**FIGURE 2:**
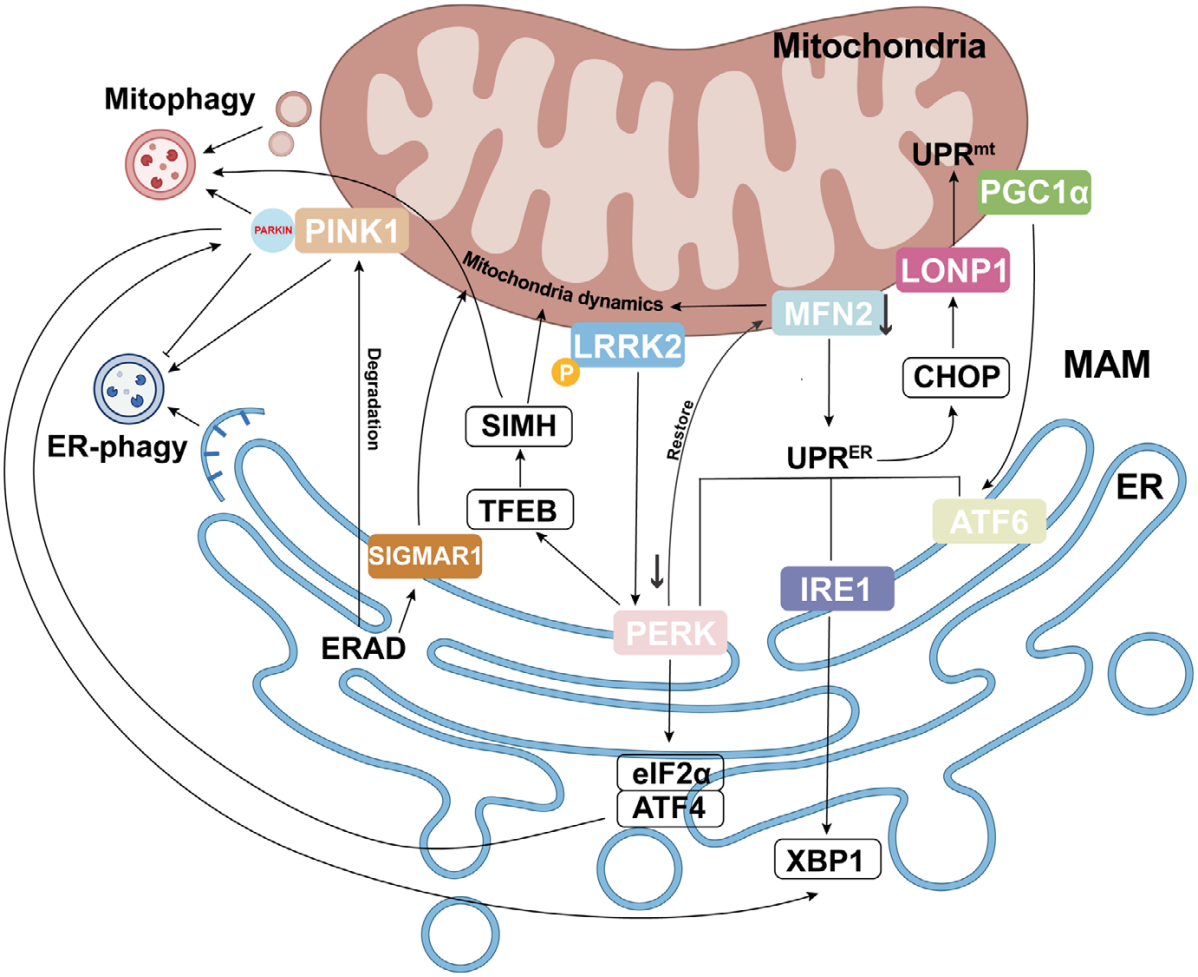
The crosstalk of QCS between mitochondria and endoplasmic reticulum (ER). The mitochondria‐associated membrane (MAM) and its dynamic interactions play a crucial role in coordinating the crosstalk between mitochondria and the ER. Key MAM molecules, including PINK1, Parkin, MFN2, LRRK2 and PGC1α, are integral to linking mitochondrial dynamics with UPR^ER^. Under stress conditions, UPR^ER^ influences the response of UPR^mt^, primarily through the PERK‐eIF2α‐ATF4 pathway and LONP1. PERK can also induce stress‐induced mitochondrial hyperfusion (SIMH) to promote mitochondrial elongation, biogenesis and mitophagy. Additionally, ERAD may modulate MAM and mitochondrial dynamics via Sigmar1. Another notable interaction pertains to the communication of selective autophagy, regulating by PINK1 and Parkin. These crosstalks highlight the essential role of MAM in maintaining cellular health.

As metabolism and synthesis centers, mitochondria and the ER are the most crucial organelles in cells and have multiple contact sites with other organelles. Their balance and coordinated interplay are fundamental for the normal cellular activities (Figure 2). As mentioned before, the MAM serves as a crucial mitochondria‐ER contact site for crosstalk between these two organelles. A growing number of molecules have been identified at MAM, such as PTEN‐induced kinase 1 (PINK), mitofusin 2 (MFN2), and phosphofurin acidic cluster sorting protein 2 (PACS2), while several molecules link MQC and ERQC (Bhat et al., 2017; Saito & Imaizumi, 2018).

MFN2, a key protein in mitochondrial dynamics, modulates the UPR^ER^ through interaction with PERK. Deletion of MFN2 disrupted the cell response to ERS and overactivated three UPR^ER^ pathways, while removal of PERK restored mitochondrial morphology in MFN2‐deficient cells (Muñoz et al., 2013; Zhang, Wu, et al., 2023). In addition, leucine‐rich repeat kinase 2 (LRRK2), commonly implicated in familial Parkinson's disease (PD), links mitogenesis and the UPR^ER^. Autophosphorylated LRRK2 stimulates PERK‐induced phosphorylation of Parkin and subsequent ubiquitination and degradation of MFN2, resulting in a reduction in MAM (Toyofuku et al., 2020). Other UPR transducers, such as ATF4, can influence MQC by modulating Parkin, which controls mitochondrial dynamics and mitophagy. Conversely, Parkin can also activate UPR^ER^. Furthermore, PGC1α, a mitochondrial biogenesis regulator, exhibits tissue‐specific connections with the UPR^ER^. In skeletal muscle, PGC1α expression is heightened during ERS, facilitating cooperation with ATF6 to orchestrate an adaptive stress response (Senft & Ronai, 2015; Wu et al., 2011).

Under stress, the UPR^ER^ may affect the UPR^mt^, such as the PERK‐eIF2α‐ATF4 signaling pathway, which governs the proteostasis and function of mitochondria (Bouman et al., 2011; Dlamini et al., 2021; He et al., 2019; Rainbolt et al., 2014). Located at MAM, PERK establishes a critical axis that senses stress in both mitochondria and ER. During ERS, PERK induces dynamic mitochondrial elongation, known as stress‐induced mitochondrial hyperfusion (SIMH), via TFEB activation and translocation to the nucleus and promotes mitochondrial biogenesis and mitophagy to prevent premature mitochondrial fragmentation (Almeida et al., 2022; Lebeau et al., 2018; Perea et al., 2023). Additionally, Lon protease (LONP1), an AAA+ ATPase in the UPR^mt^, can also participate in the signaling interplay between the UPR^mt^ and UPR^ER^. Activation of the eIF2α‐ATF4 pathway stimulates CCAAT/enhancer‐binding protein (C/EBP) homologous protein (CHOP), which subsequently enhances the expression and import of LONP1 to moderate the UPR^mt^ (Yang et al., 2018). The reciprocal activation is exemplified by Parkin to selectively modulate UPR^ER^ signaling through IRE1α/X‐box binding protein 1 (XBP1) (Chiu et al., 2023; Duplan et al., 2013).

Using 3D imaging, the generation of “megamitochondria” with abnormal MAMs in Sel1L‐Hrd1 ERAD brown adipocytes was investigated. ERAD deficiency affects MAM and mitochondrial dynamics by regulating sigma receptor 1 (Sigmar1) in MAM, revealing a new relationship between mitochondria and ER (Zhou et al., 2020). Recent studies have also revealed specialized QCS affects the mitochondrial functions through regulating the “translocase of the outer membrane” (TOM complex). An ERAD protein, Ub‐like‐domain‐containing protein 2 (UBX2), binds to TOM and engages the AAA+ ATPase Cdc48 to initiate ERAD. This novel process is known as mitochondrial protein translocation‐associated degradation (mitoTAD) (Mårtensson et al., 2019).

Another notable interplay between mitochondria and ER involves in the regulation of selective autophagy. Autophagic clearance differs between mitochondria and ER within individual cells of the Drosophila intestine. Although both organelles rely on common autophagy genes for organellar clearance, distinct receptors are employed for their removal (Li et al., 2021). PTEN‐induced kinase 1 (PINK1) and the downstream Parkin are shown to participate in mitophagy (Barazzuol et al., 2020). Recent work shows that PINK1 can trigger ER‐phagy, whereas Parkin has the opposite effect (Gelmetti et al., 2017; Li et al., 2019). Besides, collaboration between PINK1 and Keap1 is essential for the ubiquitination of the ER‐phagy receptor Rtnl1 (Wang et al., 2023). Nonetheless, the mitochondrial PINK1 content can be regulated by ERAD. To maintain low PINK1 levels, degradation occurs through the proteasome by the AAA+ ATPase segregase valosin‐containing protein (VCP) and ERAD components such as GP78 and 3‐hydroxy‐3‐methylglutaryl‐coenzyme A (HMG‐CoA) reductase degradation 1 (HRD1) (Guardia‐Laguarta et al., 2019). These findings indicate the importance of mitochondria‐ER interactions in selective autophagy to eliminate damaged proteins and organelles, thereby preserving cellular health.

Of note, small ubiquitin‐like modifiers (SUMOs) possess the capability to modulate the crosstalk between mitochondria and ER through the deSUMOylating enzyme SENP3. In models of ischemia, the UPR^ER^ triggered by PERK and cathepsin B can degrade SENP3, resulting in an upsurge of SUMOylated proteins. The deSUMOylation of Dynamin‐related protein‐1 (Drp1), a pivotal regulator of mitochondrial fission, attenuates the release of cytochrome c and mitigates caspase‐mediated cell death. Thus, the SUMOylation of Drp1 serves as an additional regulatory mechanism within the pro‐survival signaling cascade from the ER to mitochondria (Guo et al., 2013).

### 3.2 Crosstalk between mitochondria and ribosomes

**FIGURE 3:**
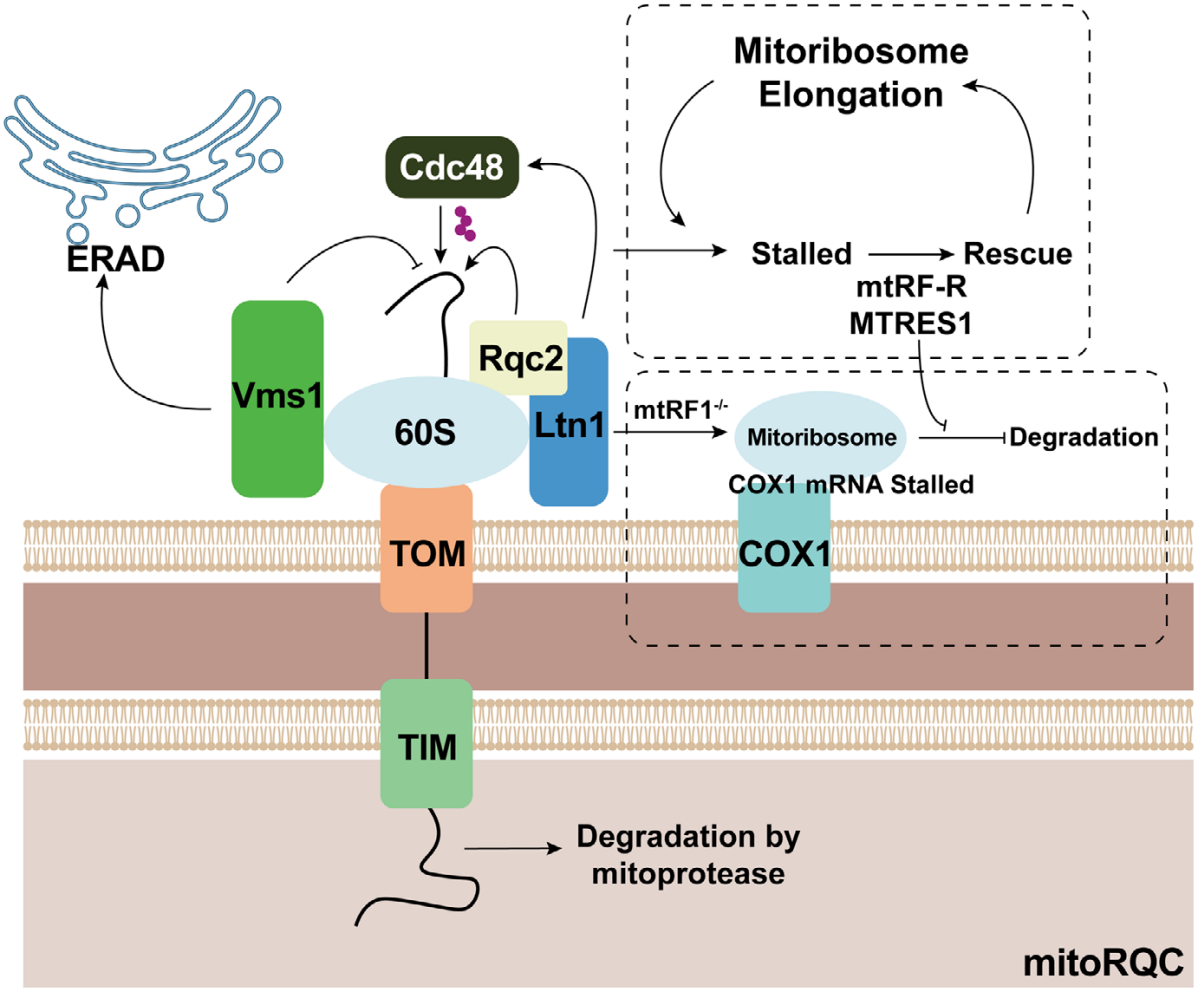
The crosstalk of QCS between mitochondria and ribosome. During synthesis, polypeptides destined for mitochondria that stall on ribosomes are tagged with a C‐terminal alanine and threonine tail (CAT‐tail) by Rqc2. These CAT‐tailed polypeptides evade ubiquitination by Ltn1 and are still imported into mitochondria, where they promote aggregation and cellular toxicity. By preventing Rqc2‐dependent CAT‐tailing, Vms1 binds to 60S ribosomes and mitigates protein aggregation and directs them to mitoRQC. Vms1 is also crucial for ERAD, and yeast cells lacking Vms1 or Cdc48 display UPS deficiencies. Additionally, stalled mitoribosomes during elongation are resolved by release factor homolog mtRF‐R and RNA‐binding protein MTRES1, which remove nascent peptides and peptidyl tRNA. Notably, mtRF1 is vital for COX1 translation termination; its loss causes COX1 deficiency, while mitoRQC activation can partially rescue stalled ribosome complexes.

Research indicates that RQC also plays a role in mitochondrial proteostasis (Figure 3). Normally, Rqc2 is recruited to the stalled 60S ribosomal subunit, where it subsequently engages with Ltn1. Then Ltn1 tags the stalled polypeptides with ubiquitin for degradation, such as cell division control protein 48 (Cdc48) (Mizuno et al., 2021). On the other hand, during synthesis, polypeptides destined for mitochondria that stall on ribosomes are tagged by Rqc2 with a C‐terminal alanine and threonine tail (CAT‐tail). These CAT‐tailed polypeptides are largely resistant to ubiquitination via the Ltn1‐dependent pathway and are subsequently imported into mitochondria, where the CAT tails promote aggregation, leading to cellular toxicity. In such cases, Vms1 (ANKZF1 in humans), a tRNA hydrolase, binds to 60S ribosomes, cleaves ribosome‐stalled mitochondrial polypeptides, and releases them into the mitochondria for protease degradation (Izawa et al., 2017; Lamech & Haynes, 2017). And by preventing Rqc2‐dependent CAT‐tailing, Vms1 mitigates the aggregation of these proteins and directs them towards mitochondrion‐localized ribosome‐associated quality control (mitoRQC) (Verma et al., 2018). Vms1 is also a key regulator of ERAD. Yeast cells deficient in Vms1 or unable to bind Cdc48 exhibit deficiencies in UPS, underscoring a mitochondrial proteostasis mechanism analogous to ERAD (Howard, 2021). Nonetheless, the precise interaction between Vms1 and Rqc2 requires further elucidation (Lenkiewicz et al., 2021; Zurita Rendón et al., 2018).

The mitochondrial ribosome (mitoribosome) and its associated proteins are involved in synthesizing subunits of oxidative phosphorylation complexes (Koripella et al., 2020). Recent studies have reported that large mitoribosomal subunits become trapped during ribosome rescue in the interrupted elongation stage. The release factor homolog C12orf65 (mtRF‐R) and the RNA‐binding protein C6orf203 (MTRES1) are involved in removing the nascent peptide and peptidyl tRNA from these stalled ribosomes (Desai et al., 2020). Later, studies determined that mtRF1 is essential for mitochondrial function, particularly in the termination of COX1 translation. While the loss of mtRF1 leads to COX1 deficiency, activation of mitoRQC can partially rescue ribosome complexes stalled in the process of translating COX1, thereby averting respiratory failure (Krüger et al., 2023; Nadler et al., 2022).

### 3.3 Crosstalk between mitochondria and the Golgi

GDVs are integral to mitochondrial fission, Golgi‐ER communication, and protein degradation to lysosomes (Chang & Yang, 2022; Rasmussen et al., 2020). Trans‐Golgi network (TGN) vesicles, containing phosphatidylinositol 4‐phosphate (PI4P) from the Golgi, are recruited to MAM to activate Drp1, which may trigger the final steps of mitochondrial division. This process is driven by ADP‐ribosylation factor 1 (Arf1) and phosphatidylinositol 4‐kinase‐III‐b (PI(4)KIIIb). Researches show that inhibiting Arf1 and PI4KIIIβ leads to elongated mitochondria with extensive interconnection and segmentation (Nagashima et al., 2020; Tábara et al., 2021). Additionally, GDVs transport proteins to the ER and plasma membrane through ERAD and the ART‐Rsp5 ubiquitin ligase network in yeast cells (Zhao et al., 2013). Nevertheless, the exact regulatory mechanisms governing this network are still unknown.

### 3.4 Crosstalk between mitochondria and peroxisomes

Crosstalk between mitochondria and peroxisomes is also significant. As previously mentioned, the biogenesis of peroxisomes could be derived from mitochondria. MDVs bearing PEX3 and PEX14 merge with vesicles derived from ER. This fusion allows newly formed peroxisomes to acquire membranes from other organelles, thereby augmenting their connectivity with mitochondria and ER (Sugiura et al., 2017). Another pathway for peroxisome formation involves elongation and division. The elongation and subsequent segregation of mature peroxisomes are facilitated by the collaboration between PEX11 and mitochondrial Fission 1 (Fis1) and mitochondrial fission factor (Mff) via recruiting dynamin1‐like (DNM1L) to sever the peroxisomal membrane, thereby facilitating division (Koch & Brocard, 2012).

Besides, the OMM‐anchored BCL2‐interacting protein 3‐like (BNIP3L)/NIX protein, as a receptor for mitophagy, autonomously localizes to peroxisomes and induces pexophagy. Thus, pexophagy is triggered under physiological circumstances that also initiate mitophagy, such as the differentiation of cardiomyocytes and erythrocytes (Wilhelm et al., 2022).

### 3.5 Crosstalk between mitochondria and LD

The LD‐associated mitochondria, known as peridroplet mitochondria (PDM), are distinct from cytoplasmic mitochondria (CM) and are crucial for lipid metabolism (Benador et al., 2018; Song et al., 2023). PDMs are regulated by perilipin 5 (PLIN5), a protein abundantly expressed on LD surfaces. Studies have found that disruptions in mitochondrial dynamics may impair the functions of both PDM and CM. Consequently, a reduction in PDM can hinder mitochondrial dynamics due to decreased MFN2 expression, which directly interacts with PLIN1. Thus, PDM serves as a vital connection between impaired mitochondrial dynamics and metabolic dysregulation (Benador et al., 2019; Boutant et al., 2017).

### 3.6 Crosstalk between the ER and ribosome

**FIGURE 4:**
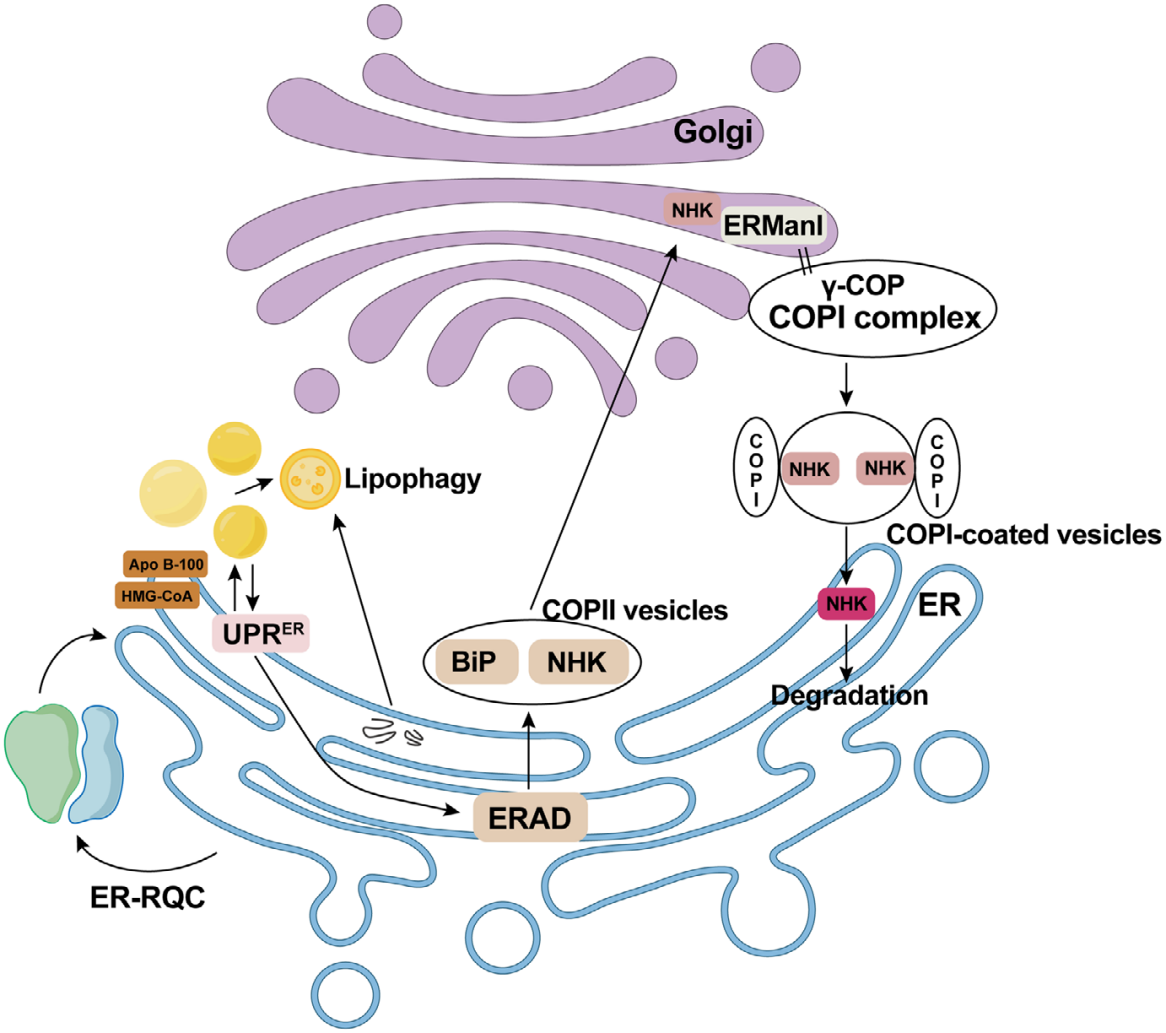
The crosstalk of QCS between the ER and other organelles. ER and Ribosome crosstalk: The interplay between ERQC and RQC is integral for protein homeostasis. RQC supports protein quality in both co‐translational and post‐translational pathways, with ER‐RQC specifically regulating membrane protein synthesis at the ER membrane. ER and Golgi crosstalk: QCS between the ER and Golgi ensure proper protein folding and transport. BiP is transported to the Golgi via a COPII‐dependent pathway while ERManI and γ‐COP facilitate the removal of ERAD regulators, such as NHK, from the Golgi via COPI‐coated vesicles. ER and Lipid Droplet (LD) Crosstalk: The balance between ER and LDs impacts their proteome compositions. UPR^ER^ activation induces LD biogenesis, while lack of LD formation enhances UPR pathways. Besides, LDs interact with ERAD substrates like Apo B‐100 and HMG‐CoA before their proteasomal degradation. LDs may also participate in microlipophagy for ER protein degradation with unclear mechanisms.

The interplay between ERQC and RQC remains underexplored while evidence suggests a cooperative engagement between the two in regulating protein quality control (Figure 4). For example, RQC performs to preserve protein homeostasis in both post‐translational and co‐translational pathways. The latter is intricately associated with ER membrane for the synthesis of membrane proteins (MPs), which is defined as ER‐associated RQC (ER‐RQC) (Arakawa et al., 2016; Phillips & Miller, 2020). And AGOs can also engage in posttranscriptional miRNA‐mediated gene silencing with ER, thereby modulating RQC through facilitating Ltn1 (Gao, Zhu, et al., 2023).

### 3.7 Crosstalk between ER and the Golgi

The QCS between ER and Golgi interact in several ways to ensure proper protein folding and transport. Early in 2006, Pimpl et al. demonstrated that BiP, an ERAD factor, is transported to the Golgi‐mediated vacuole by coat protein complex II (COPII)‐dependent pathway in tobacco (Pimpl et al., 2006). Later, in mammalian cells, it was discovered that ER α‐1, 2‐mannosidase (ERManI) and γ‐COP could help to clear another regulators of ERAD, null Hong Kong (NHK), from a Golgi‐based QCS for returning ERAD components back to ER through COPI‐coated vesicles (Pan et al., 2013). Notably, some proteins, such as Wsc1p, undergo rapid degradation by Golgi quality control rather than ERAD, suggesting a potential crosstalk between ER and Golgi QCS (Wang & Ng, 2010).

### 3.8 Crosstalk between the ER and LD

As derived from ER, the LD‐ER balance maintains the proteome compositions of both ER and LDs. Activation of UPR^ER^ can trigger LD biogenesis, while the absence of LD biogenesis can, in turn, enhance UPR pathways (Ben M'barek et al., 2017). Studies have shown that LDs temporarily interact with apolipoprotein B‐100 (Apo B‐100) and HMG‐CoA reductase, two ERAD substrate proteins, before their proteasomal degradation, which is related to lipid metabolism (Stevenson et al., 2016). Additionally, LDs may assist in the degradation of ER protein by microlipophagy, though the exact substrate proteins still need to be identified (Garcia et al., 2018).

### 3.9 Crosstalk between lysosomes and other organelles

Lysosomes can directly interact with other organelles as they participate in the terminal step of selective autophagy of cellular organelles mentioned above. Beyond their degradation ability, lysosomes are now recognized for their roles in connecting with other cellular organelles via MCS (Henne, 2021). It has been delicately documented that MCS between lysosomes and mitochondria can assist the bidirectional crosstalk, including mitochondrial fission, lysosomal dynamics, and lysosomal degradation of MDVs, mainly through Rab7 GTP hydrolysis (Wong et al., 2018, 2019). Recently, the induction of PINK1/Parkin‐mediated mitophagy is shown to be involved in lysosomal biogenesis by promoting TFEB translocation and expression. Conversely, upregulation of TFEB stimulated mitochondrial biogenesis with elevated level of PGC1α (Ivankovic et al., 2016; Malik et al., 2023). Additionally, mitochondria‐lysosome contact sites can also simultaneously engage with the ER, supporting integrated regulation of organelle homeostasis (Rowland et al., 2014; Wong et al., 2019).

## 4 SUMMARY OF DYSFUNCTIONAL QCS DURING AGING

### 4.1 MQC and aging

Over the past decades, studies have shown that damage to the mitochondria during aging, including mtDNA mutations, excessive oxidative stress, proteome instability, and reduced mitochondrial biogenesis, can induce a series of MQC to maintain cellular homeostasis (Eldeeb et al., 2022; Roque et al., 2020).

In particular, the progressive accumulation of misfolded proteins such as tau, amyloid precursor protein (APP), and α‐synuclein caused by dysregulation of RQC initiates UPR^mt^ and elicits cellular stress in neurodegenerative diseases (Gao et al., 2024; Guo et al., 2023). Under such stress, mitophagy and MDVs are activated to mitigate mitochondrial dysfunction. Nevertheless, aging impairs mitophagy due to mutations in PINK1 and Parkin, and mitochondria in older PD patients exhibit inadequate MDVs secretion (Eldeeb et al., 2022; Grossmann et al., 2023). Furthermore, the age‐related mtDNA damage in lung diseases tends to upregulate mitochondrial fusion‐related proteins such as MFN1 and optic atrophy‐1 protein (OPA1) to compensate mitochondrial deficiencies (Liu et al., 2023).

Notably, recent findings suggest that these MQC may accelerate aging in turn, which indicates that MQC is a double‐edged sword, either prolonging or expediting the aging process (Guo et al., 2023).

### 4.2 ERQC and aging

Similar to the activation of UPR^mt^ by misfolded proteins during aging, these aberrant proteins caused by multiple factors, such as genetic mutation, oxidative stress, and inflammation, can also induce ERS and UPR^ER^. The prolonged activation or dysregulation of the ERS and UPR^ER^ levels exacerbate cellular stress and even lead to cell death that contributing to conditions including neurodegeneration, diabetes, atherosclerosis, and cancer (Celik et al., 2023; Ghemrawi & Khair, 2020; Ochoa et al., 2018). In neurodegenerative disorders, mutant superoxide dismutase 1 (SOD1) and huntingtin can inhibit ERAD through abnormal protein–protein interactions. Meanwhile, the Sel1L‐Hrd1 ERAD complex may influence tumorigenesis by regulating ER homeostasis (Hetz & Saxena, 2017; Kim et al., 2015). The exact role of ER‐phagy during aging remains unclear. However, mutations in certain ER‐phagy receptors have been associated with neurodegeneration, diabetic kidney disease, intervertebral disc degeneration, and cancers (Cherubini & Zito, 2022; Luo et al., 2022; Yang et al., 2023).

### 4.3 Other QCS and aging

As previously mentioned, compromised RQC functions result in the aggregation of defective proteins and increased proteotoxic stress, which has been observed in several aging‐related diseases. Conversely, the prolonged existing toxic proteins and mutations can exacerbate ribosomal stalling, thereby further compromising RQC (Gao et al., 2024; Martin et al., 2020).

Peroxisomal metabolic dysfunction is implicated in neurodegeneration. Jo, et al. showed that the depletion of HSPA9, a new pexophagy regulator, inhibited peroxisomal biogenesis while elevating peroxisomal ROS and pexophagy. And the HSPA9 mutants were found in PD, which failed to reverse these effects (Jo, Park, & Cho, 2020; Jo, Park, Kim, et al., 2020). Furthermore, LDQC, such as lipophagy, fundamentally regulates cell death and enhances metabolism of toxic lipids. In pathologic conditions, overactivated lipophagy accelerates the LD catabolism and hepatic stellate cells (HSC) differentiation to promote liver fibrosis and tumor growth (Filali‐Mouncef et al., 2022; Zhang et al., 2022).

**FIGURE 5:**
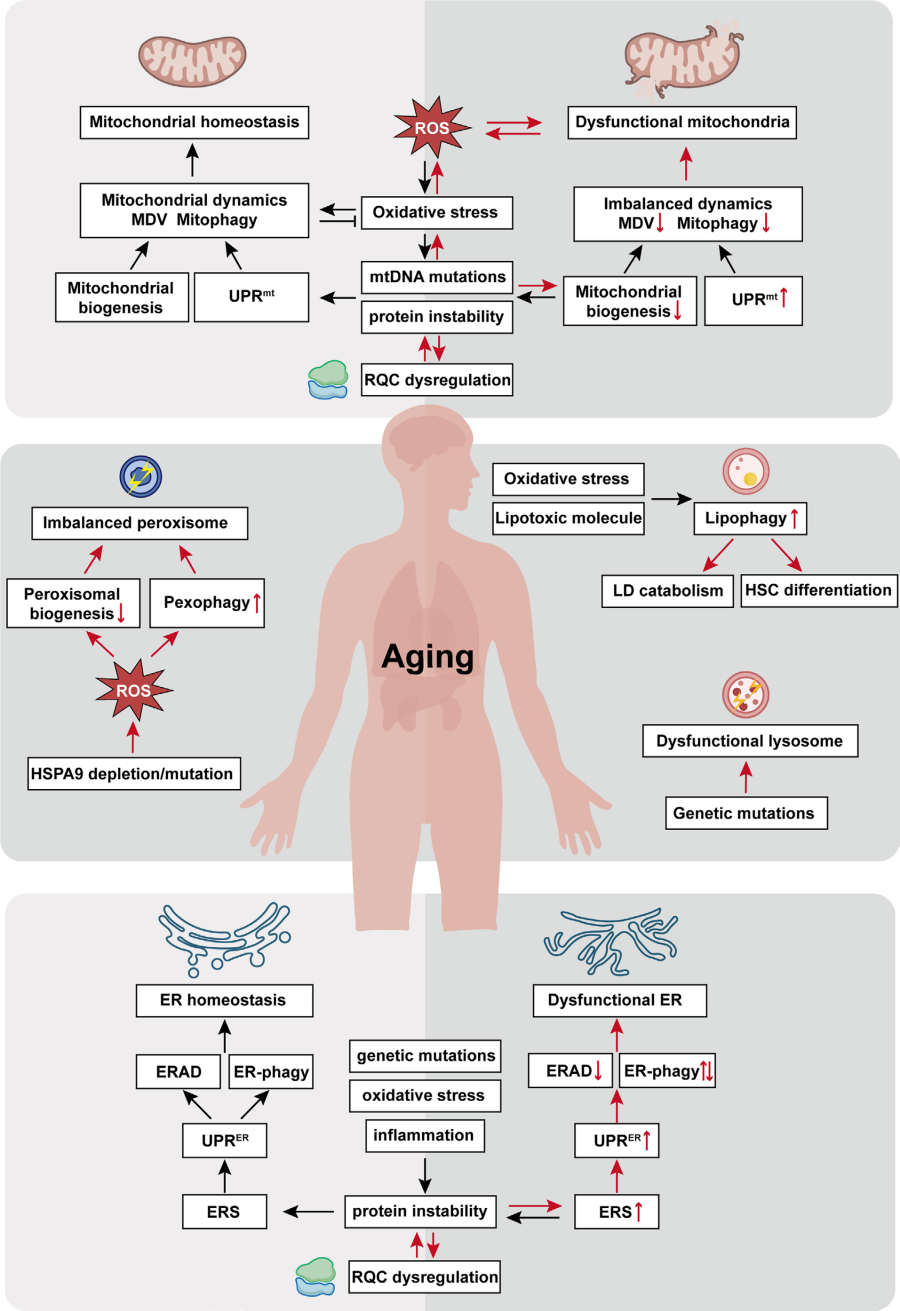
Summary of dysfunctional QCS during aging. Aging disrupts quality control systems, affecting cellular function and contributing to aging‐related diseases. Mitochondrial and ER dysfunction, marked by genetic mutations and oxidative stress, impairs their quality control process. This leads to UPR^mt^ and UPR^ER^, exacerbating conditions like neurodegenerative diseases, diabetes, and cancer. Additionally, compromised ribosome quality control results in increased proteotoxic stress. Peroxisomal, lysosomal and lipid droplet dysfunction further aggravates aging‐related pathologies. Understanding these dysregulated systems is crucial for addressing their roles in aging and developing targeted interventions.

Importantly, dysfunction of lysosomes is also linked to neurodegenerative events, as mutations in genes that related to the lysosome quality control are identified (Griffey & Yamamoto, 2022). Despite increasing evidence highlighting impaired QCS during aging and aging‐related diseases, the complex landscape of this field still needs to be fully explored (Figure 5).

## 5 DEFICIENCIES IN ORGANELLAR QUALITY CONTROL CROSSTALK IN AGING‐RELATED DISEASES

### 5.1 Neurodegenerative disease

**FIGURE 6:**
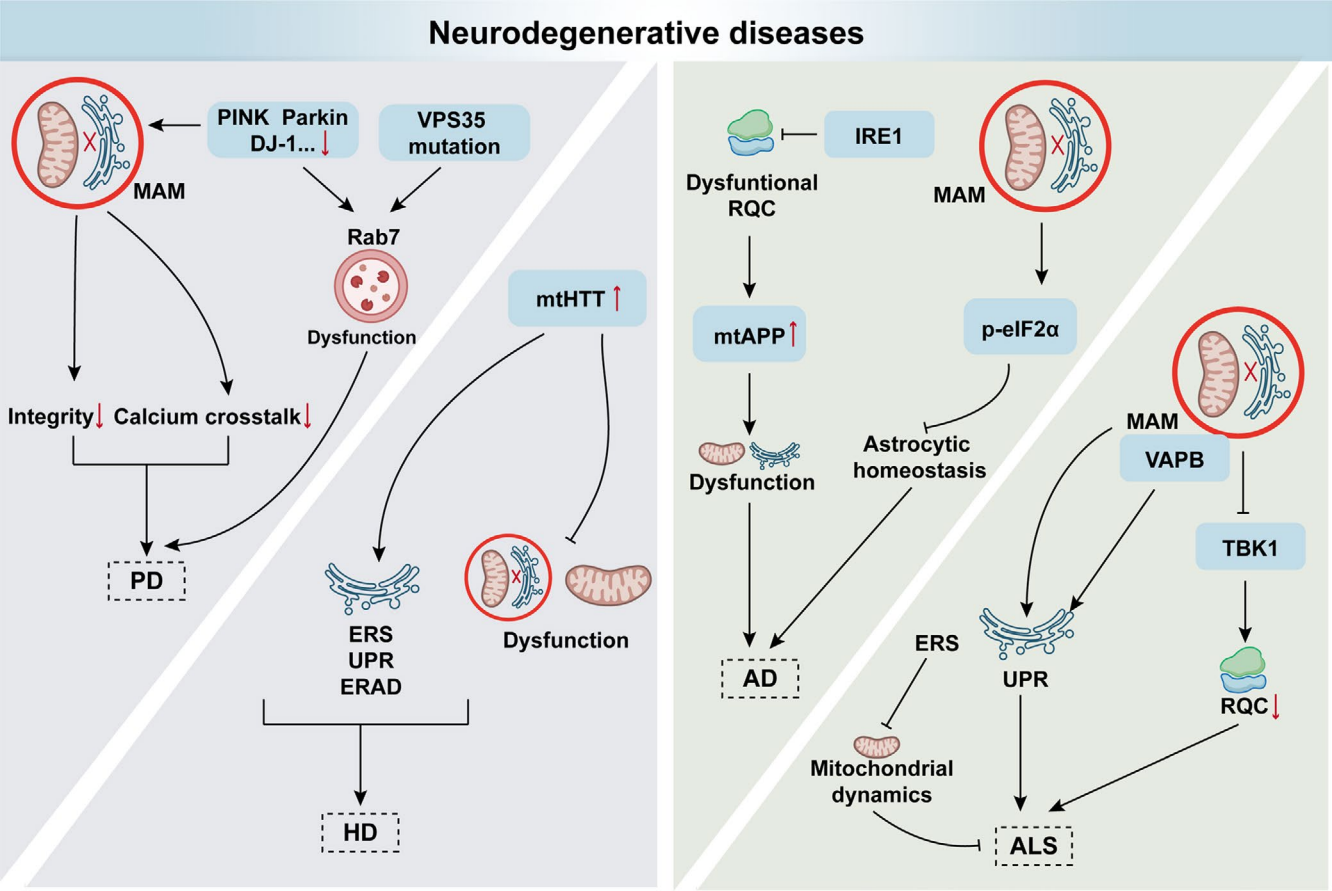
Dysfunctional QCS crosstalk in neurodegenerative diseases. In Parkinson's disease (PD), mitochondrial quality control is disrupted by defects in PINK1, Parkin, and DJ‐1, which impair mitochondrial‐ER interactions. And VPS35 and Parkin mutations dysregulate Rab7, thus impairing lysosomal homeostasis in PD pathogenesis. Huntington's disease (HD) involves mutant huntingtin (mtHTT) and disturbed protein homeostasis, affecting mitochondrial dynamics and ER quality control. Alzheimer's disease (AD) features amyloid precursor protein (APP) perturbations and compromised protein quality control, with RACK1 and IRE1 influencing APP processing and phosphorylated eIF2α impacting astrocytic homeostasis. Amyotrophic lateral sclerosis (ALS) shows disruptions in MAM and ERS. Depletion of MAM proteins and mutated VAPB lead to UPR^ER^ activation and degradation of RQC proteins, thus contributing to motoneuron degeneration.

Neurodegeneration associated with neuroinflammation is marked by compromised mitochondrial and lysosomal functions, while emerging evidence shows that QCS crosstalks contribute to the progression of neurodegenerative diseases (Figure 6) (Gao, Jiang, et al., 2023; Gao, Zhu, et al., 2023; Park et al., 2018).

The pathogenesis of Parkinson's disease (PD) is strongly due to a disturbance in MQC related to PINK1/Parkin and lysosomal dysfunction. Several mutations linked to familial PD, such as vacuolar protein sorting 35 (VPS35) and Parkin, are prone to dysregulate Rab7, underscoring a potential role for impaired mitochondria‐lysosome contact in PD development (Song et al., 2016). In addition, the autosomal recessive early‐onset PD is linked to mutations in DJ‐1. Liu et al. demonstrated that DJ‐1 interacted with IP3R3‐Grp75‐VDAC1 complexes at MAMs, which regulate the integrity and calcium crosstalk between mitochondria and ER. Reduced DJ‐1 levels in sporadic PD patients' substantia nigra correlate with reduced IP3R3‐DJ‐1 interaction and impaired MAM, contributing to PD pathogenesis (Gómez‐Suaga et al., 2018; Liu et al., 2019). Huntington's disease (HD) is a worldwide autosomal dominant neurodegenerative disease characterized by motor and cognitive impairments, resulting from a mutant huntingtin (mtHTT) protein (Tabrizi et al., 2022). The pathogenesis of HD is multifaceted, and recent studies suggest that the disruption in protein homeostasis, involving ERS and subsequent UPR and ERAD, participate in the HD development. Beyond these mechanisms, mitochondrial dynamics and MAM also contributes with the involvement of proteins such as MFN2 and Sigmar1 (Brondani et al., 2023; Maity et al., 2022). Although direct evidence is currently lacking, the aforementioned findings indicate that the interplay between mitochondria and ER play a crucial role in the development of these aging‐related diseases.

Alzheimer's disease (AD) is a progressive neurodegenerative disorder with perturbations in the amyloid precursor protein (APP). Protein RQCs are involved in removing defective proteins to maintain cellular proteostasis. It has been shown that in AD, disruptions in protein synthesis, mediated by phosphorylation of eIF2α, impact astrocytic homeostasis, likely due to altered MAM distance. This may result in impaired secretion of neurotrophic and neuroprotective molecules and extracellular matrix. The 4‐phenylbutyric acid (4‐PBA) rescues protein synthesis and p‐eIF2α levels, indicating its therapeutic potential (Tapella et al., 2022; Vaillant‐Beuchot et al., 2021). Moreover, mutant APP exhibited anomalous ER and mitochondrial morphology, deficient mitophagy, impaired locomotion, and reduced longevity. Genetic removal of the RQC factor RACK1 reduced mutant APP levels, while RACK1 overexpression increased them. Additionally, the ERS regulator IRE1 reduced mutant APP level through the RQC‐associated ATPase VCP/p97 and Hrd1, which also regulate wild‐type APP. These findings highlight RACK1 and IRE1 roles in APP quality control and their implications for AD treatment (Li et al., 2024).

Amyotrophic lateral sclerosis (ALS) is a fatal neurodegenerative ailment marked by the specific demise of motor neurons. A previous study reported that MAM disruption is a prevalent pathology in Sigmar1‐ and SOD1‐linked ALS (Watanabe et al., 2016). MAM can activate TANK‐binding kinase 1 (TBK1) during proteostatic strain, with the MAM‐specific E3 ubiquitin ligase AMFR catalyzing the ubiquitination of nascent proteins to activate TBK1, thereby prompting degradation of ribosomal proteins. Deficiency in MAM or TBK1 heightens cellular susceptibility and motor dysfunction, aggravating proteostatic stress in ALS (Watanabe et al., 2023). Moreover, several studies have revealed the induction of ERS and UPR^ER^ in ALS, presumed to be pivotal in motoneuron degeneration. Depletion of MAM proteins (MFN2, PACS2, Sigmar1) and the expression of mutated vesicle‐associated membrane protein (VAMP)‐associated protein B (VAPB) induce the UPR^ER^ by interacting with UPR sensors, including ATF6, IRE1, and PERK. Moreover, alleviating ERS with salubrinal, a selective inhibitor of eIF2α dephosphorylation, rescues mitochondrial dynamics and prevents motoneuron degeneration (Bernard‐Marissal et al., 2015, 2018).

In fact, VAPB, involving in coatomer‐mediated ER‐Golgi transport, has been detected in patients with familial ALS. VAPB serves as a tether at MAM by recognizing a specific motif containing two phenylalanines (FFs) within an acidic tract (FFATs). This pattern enables the major sperm protein (MSP) domain of VAPB to establish a scaffold between ER and neighboring organelles hosting an FFAT protein (Gómez‐Suaga et al., 2019; Markovinovic et al., 2024). Therefore, VAPB has the ability to regulate various functions in CNS cells, including protein conveyance, lipid metabolism, UPR, and mitophagy. Mutant VAPB can induce autophagy, leading to the accumulation of dysfunctional mitochondria, a process possibly exacerbated by mutations in optineurin, an autophagy receptor for impaired mitochondria (Chen et al., 2021; Li et al., 2021; Markovinovic et al., 2022; Parakh & Atkin, 2021; Toth & Atkin, 2018).

### 5.2 Pulmonary disease

Chronic obstructive pulmonary disease (COPD) ranks among the most prevalent pulmonary diseases affecting elderly populations globally. It is distinguished by persistent airflow restrictions due to prolonged inflammation. However, the exact mechanisms driving this pathology remain elusive (Christenson et al., 2022). Dysfunction of multiple organelles, including mitochondria, ER, Golgi, and lysosomes, contributes to COPD. Of note, impaired PINK1/Parkin signaling pathway‐dependent mitophagy induces ROS and inflammasome production in small airway epithelial cells, which significantly accelerates cellular senescence in response to cigarette smoke exposure and exacerbates pathological changes in lung tissues (Araya et al., 2019; Ito et al., 2015).

Recently, mitochondria‐ER crosstalk has been reported to be disrupted in idiopathic pulmonary fibrosis (IPF), a progressive parenchymal lung condition with unidentified etiology (Aghaei et al., 2020; Manevski et al., 2020). In alveolar epithelial cells (AECs), ATF4 is recognized as a central regulator of the UPR^mt^ in response to various mitochondrial stressors, and the UPR^ER^ activates the UPR^mt^, leading to mitochondrial dysfunction through an ATF4‐dependent mechanism. In the lungs of bleomycin‐treated aged mice and IPF patients, elevated alveolar epithelial ATF4 aggravates pulmonary UPR^mt^, lung inflammation, and even death, which indicates that ERS and consequent mitochondrial dysfunction contribute to the development of a variety of pulmonary diseases (Bao et al., 2024; Jiang et al., 2020). In addition, knockout of PINK1 in alveolar type 2 AECs leads to an abnormal mitochondrial morphology and an increased susceptibility to fibrotic lung remodeling following viral infection, while changes in PINK1 expression and mitophagy are observed in IPF type 2 AECs (Bueno et al., 2015; Katzen & Beers, 2020). The ERS protein ATF3 acts as a suppressor of PINK1. Mice harboring a conditional deletion of ATF3 in type 2 AECs demonstrate resilience against bleomycin‐induced fibrosis, implying the association among ERS, mitochondrial dysfunction, and fibrotic remodeling (Bueno et al., 2018). However, the interplay between different QRCs and their pathologic implications in chronic lung diseases remains unclear.

### 5.3 Cardiometabolic disease

**FIGURE 7:**
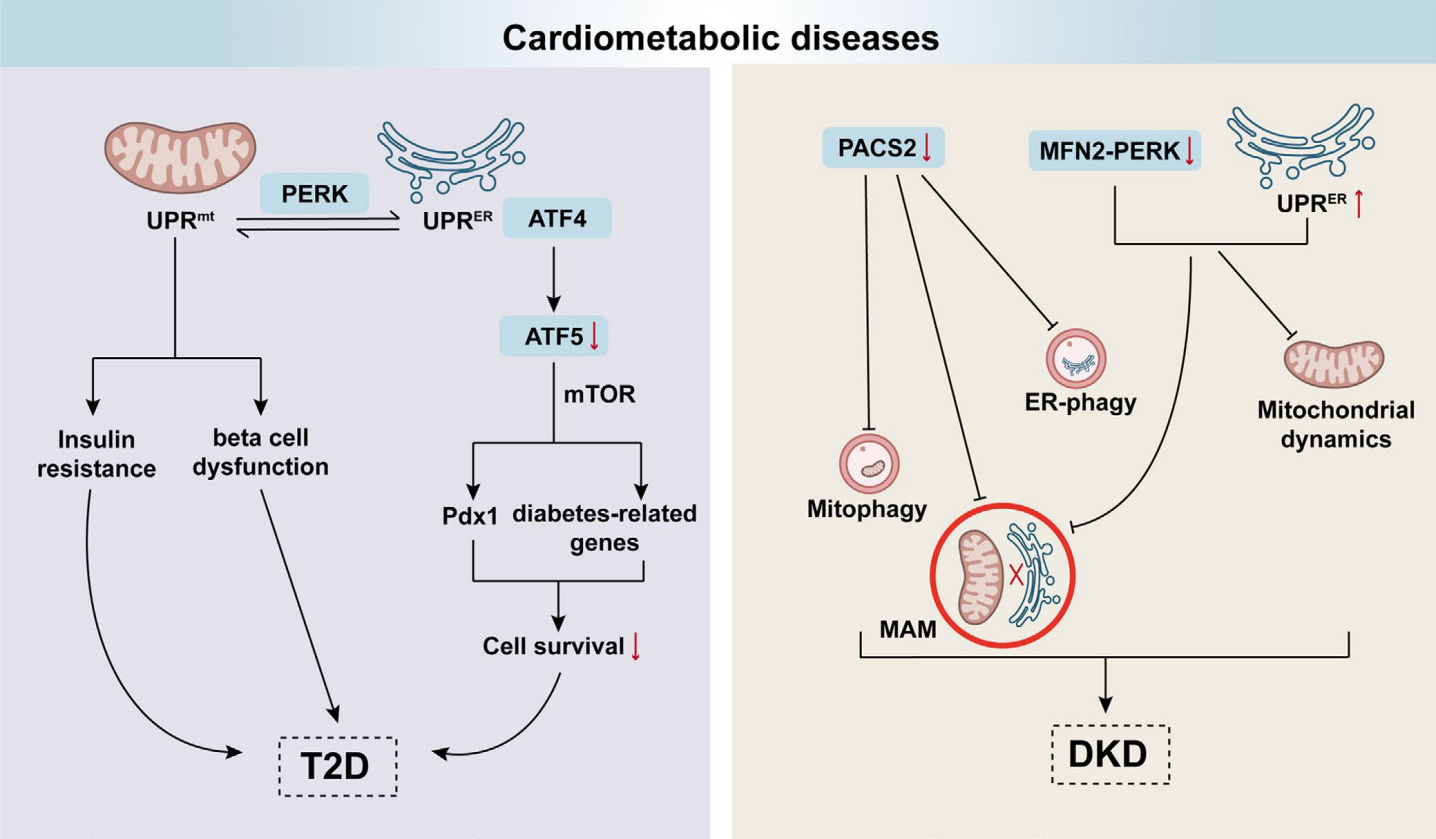
Dysfunctional QCS crosstalk in cardiometabolic diseases. The UPR^mt^ and UPR^ER^ are essential for cellular homeostasis and are implicated in metabolic diseases like type 2 diabetes (T2D). UPR^mt^ affects insulin resistance and beta cell function, while UPR^ER^, via the PERK pathway, increases cellular susceptibility in T2D. PACS2, a key protein at MAM, maintains mitochondrial, ER, and lysosomal balance. Its absence worsens diabetic kidney disease (DKD) by disrupting MAM formation and mitophagy. Additionally, decreased MFN2 and UPR^ER^ activation in high glucose models indicate that MFN2‐PERK signaling may be a promising target for DKD treatment.

Dysregulated homeostasis of cellular organelles is observed in cardiometabolic diseases during aging (Figure 7) (Ajoolabady et al., 2021). In particular, there has been a growing recognition of the critical role that maintaining the functional integrity of multiple cellular compartments plays in the functionality and senescence of cardiomyocytes. Deficiencies in mitophagy resulting from the deletion of PINK1 or Parkin intensify the cytotoxic response of oxidized low‐density lipoproteins on vascular smooth muscle cells. Conversely, the upregulation of PINK1 or Parkin has been demonstrated to confer protective effects against the development of atherosclerosis. Additionally, malfunctions in other cellular compartments, like ER, also contribute to the abnormal CMs and is an important factor in cardiovascular diseases (CVDs) (Yao et al., 2021).

The UPR^mt^ and UPR^ER^ are crucial mechanisms for maintaining homeostasis and are implicated in various metabolic diseases. For example, type 2 diabetes (T2D) poses a considerable global health issue, marked by peripheral tissue insulin resistance and pancreatic beta cell dysfunction. Substantial evidence indicates that the UPR^mt^ impacts insulin resistance and beta cell malfunction in T2D (Fernandes et al., 2020; Kos, 2020). Moreover, recent research suggests that the UPR^ER^ coordinates with the UPR^mt^ in T2D by activating the PERK pathway to increase the susceptibility of cells. Pancreas/duodenum homeobox protein 1 (Pdx1) and diabetes‐related genes are implicated in beta cell survival through direct regulation of ATF5, a downstream target of ATF4, modulated by eIF4E‐binding protein 1 (4ebp1) within the mammalian target of rapamycin (mTOR) pathway. The absence of ATF5 consequently reduces stress‐induced translational inhibition, thereby enhancing cell susceptibility to stress‐induced death (Kang et al., 2022).

PACS2, a multifunctional protein at MAMs, orchestrates mitochondrial, ER, and lysosomal homeostasis in conditions, such as obesity, neurodegenerative diseases, and cancer (Li et al., 2020). A previous study has shown a positive correlation between PACS2 expression and renal function and a negative correlation with the severity of tubulointerstitial lesions. Conditional deletion of PACS2 in proximal tubules (PTs) exacerbates diabetic kidney disease (DKD) by disrupting MAM formation and mitophagy (Li et al., 2022). Further research has revealed that PACS2 promotes the nuclear translocation of TFEB to activate FAM134B, which is involved in ER‐phagy (Yang et al., 2023). Therefore, these data unveil the role of PACS2 in ameliorating renal tubular injury in DKD by monitoring mitophagy and inhibiting ER‐phagy.

In addition to selective autophagy, the dysregulated interaction between mitochondrial dynamics and the UPR also results in the progression of DKD. Cao et al. documented decreased MFN2 expression and UPR^ER^ activation in high glucose (HG)‐induced diabetic models, accompanied by mitochondrial dysfunction, diminished MAM, and increased apoptosis. HG induces a decline in the MFN2‐PERK interaction, suggesting that the MFN2‐PERK signaling pathway may serve as a novel therapeutic target to prevent podocyte injury in DKD (Cao et al., 2021).

### 5.4 Cancer

Interactions between cellular organelles significantly influence the progression of multiple cancers (An et al., 2024). For instance, Wang et al. discovered that the XBP1‐membrane‐associated ring finger protein 5 (MARCH5)‐MFN2 pathway contributes to resistance against ERS by cooperating mitochondrial dynamics and mitophagy in melanoma. Activation of the IRE1α/ATF6‐XBP1 promotes transcription of MARCH5, which promotes the degradation of MFN2. This process triggers mitochondrial fission and mitophagy under ERS conditions, thereby enhancing ERS resistance in melanoma (Wang et al., 2021). The inter‐organellar communication also holds promising anti‐cancer potential (Bhat et al., 2017). Che et al. uncovered that p‐Drp1^Ser616^ anchors to mitochondria‐lysosome MSC through combination with Rab7, which stimulates PINK1/Parkin‐mediated mitophagy and mitigates apoptosis in hepatocellular carcinoma (HCC) cells undergoing chemotherapy. Furthermore, activation of B56γ can dephosphorylate pDrp1^Ser616^, thereby suppressing mitophagy, increasing mitochondrial apoptosis, and intensifying the anti‐cancer effects against HCC (Che et al., 2022). Nonetheless, more work is warranted to clarify the interaction between organellar quality control in tumorigenesis and its diagnostic and therapeutic potential.

## 6 CONCLUSIONS AND PERSPECTIVES

Pharmacological activation or inhibition of specific targets of multiple QCS in aging and aging‐associated diseases have shown promising results. For example, therapeutic approaches, such as SGLT2 (sodium‐glucose linked transporter 2) inhibitor and GLP1 (glucagon‐like peptide‐1) receptor agonists, along with exercise, can elevate MFN2 expression to enhance mitochondrial dynamics, thereby improving the function of cardiomyocytes in diabetic cardiomyopathy (Croteau et al., 2021; Zhou et al., 2023). Additionally, PERK is identified as a drug target in neurodegenerative and metabolic diseases, with its pharmacological modulation extensively characterized by Almeida et al. (Almeida et al., 2022). Despite these advancements, research on regulatory‐targeted drugs specifically aimed at QCS interactions remains limited. Notably, a study demonstrated that flavonoids from Selaginella tamariscina could mitigate doxorubicin‐induced cardiotoxicity by alleviating mitochondria dysfunction and ERS through MFN2/PERK pathway (Gao et al., 2022). Similarly, melatonin has shown to attenuate isoflurane‐induced ERS, neuroapoptosis, and neuroinflammation via SIRT1/MFN2/PERK pathway, thus improving spatial learning and memory deficiencies (Fang, Han, et al., 2022). Furthermore, AGK2, a SIRT2 inhibitor, exhibits a hepatoprotective role in acute liver failure by upregulating the expression of MFN2/PERK and ferroptosis pathway (Zhang et al., 2024). These data indicate a promising therapeutic potential in targeting intraorganellar communications in aging‐related diseases.

So far, technological approaches have greatly expanded our horizons of QCS and its role in aging‐related diseases. Breakthroughs in imaging techniques, such as cryo‐electron microscopy (cryo‐EM) and structured illumination microscopy (SIM), now allow for direct visualization of cellular processes, shedding new light on organelle dynamics (Fang, Chen, et al., 2022; Fang, Han, et al., 2022; Leisten et al., 2023). Additionally, subcellular proteomics and metabolomics have uncovered the molecular changes associated with aging at the organellar level (Thul et al., 2017). However, there are still notable gaps and limitations in the current research on QCS interactions. First, the intricate mechanisms by which QCS malfunctions contribute to aging‐related pathologies are not fully elucidated. The complexity of these QCS presents a significant challenge in identifying specific therapeutic targets. Moreover, current studies tend to focus on individual QCS pathway without sufficiently addressing the synergistic effects of their interactions. This fragmented approach limits our understanding of how these systems co‐regulate and influence each other under physiological and pathological conditions. In the future, enhanced strategies, such as improved super‐resolution live microscopy, single‐molecule super‐resolution imaging, and organellar‐based spatiotemporal proteomes, may provide further details into QCS interplay in aging‐related diseases (Leisten et al., 2023; Zhu et al., 2024).

In conclusion, this article reviews the fundamental pathways of organellar QCS and highlights recent findings regarding their potential pathophysiologic roles in aging‐related diseases, including neurodegenerative, pulmonary, cardiometabolic disorders, and cancers (Table 1). Although the complex mechanisms driving the malfunctions of QCSs in these diseases remain elusive, compelling evidence suggests that interaction among various QCS components can serve as potential diagnostic and therapeutic targets. Further investigation is warranted to deepen our understanding of the mechanism and role of QCS crosstalk as biomarkers and therapeutic agents for the management of aging‐related diseases.

**TABLE 1:** The interaction of QCS and their roles in aging‐related diseases.

Disease	QCS interaction	Protein	Mechanism
Parkinson's disease	Proposed	PINK1, Parkin, VPS35, Rab7, DJ‐1	The disturbance in MQC involving PINK1 and Parkin leads to PD. Mutations, such as VPS35 and Parkin dysregulates lysosomal Rab7. DJ‐1 interacts with IP3R3‐Grp75‐VDAC1 complexes at MAMs to maintain mitochondrial‐ER integrity and calcium signaling. Lower DJ‐1 levels reduce IP3R3‐DJ‐1 interaction and impaire MAM, contributing to PD.
Huntington's disease	Proposed	PERK, IRE1α, ATF6, MFN2, Sigmar1	Overstressed ERS, UPR, ERAD, mitochondrial dynamics and MAM disrupt protein homeostasis in HD.
Alzheimer's disease	Mitochondria‐ER	eIF2α	Altered MAM distance with elevated phosphorylated eIF2α impair protein synthesis, affecting astrocytic homeostasis.
	ER‐Ribosome	RACK1, IRE1, VCP/p97, Hrd1	RACK1 and IRE1 reduces mutant APP level via the RQC‐associated ATPase VCP/p97 and Hrd1.
Amyotrophic lateral sclerosis	Mitochondria‐ER	Sigmar1, SOD1, TBK1, MFN2, PACS2, Sigmar1, VAPB, ATF6, IRE1, PERK	MAM and TBK1 disruption increase cellular vulnerability and motor dysfunction, aggravating proteostatic stress in Sigmar1‐ and SOD1‐linked ALS.
			Loss of MAM proteins and mutated VAPB trigger UPR^ER^, while alleviating ERS rescues mitochondrial dynamics and prevents motoneuron degeneration.
	ER‐Golgi	VAPB	VAPB forms a scaffold involved in ER‐Golgi transport observed in familial ALS.
Chronic obstructive pulmonary disease	Proposed	/	Dysfunction of multiple organelles, including mitochondria, ER, Golgi and lysosomes, contributes to COPD.
Idiopathic pulmonary fibrosis	Mitochondria‐ER	ATF4, PINK1, ATF3	In alveolar epithelial cells, ATF4 regulates UPR^mt^, and the UPR^ER^ activates the UPR^mt^ and mitochondrial dysfunction via ATF4. In the lungs of mice and patients with IPF, elevated ATF4 worsens UPR^mt^, lung inflammation and mortality.
			ATF3 reduces PINK1 and leads to an abnormal mitochondrial morphology and fibrotic remodeling.
Type 2 diabetes	Mitochondria‐ER	PERK	UPR^ER^ collaborates with the UPR^mt^ in T2D by activating the PERK pathway to increase the susceptibility of cells.
Diabetic kidney disease	Mitochondria‐ER	PACS2, FAM134B, MFN2, PERK	Conditional PACS2 deletion in proximal tubules impairs MAM formation and mitophagy. PACS2 promotes the nuclear translocation of TFEB to activate FAM134B‐induced ER‐phagy.
			Decreased MFN2‐PERK interaction and UPR^ER^ activation in diabetic models, accompanied by mitochondrial dysfunction, impaired MAM and elevated apoptosis.
Melanoma	Mitochondria‐ER	XBP1‐MARCH5‐MFN2, IRE1α/ATF6‐XBP1	XBP1‐MARCH5‐Mfn2 pathway increase resistance against ERS by cooperating mitochondrial dynamics and mitophagy in melanoma. Activation of the IRE1α/ATF6‐XBP1 pathway promotes MARCH5 transcription and MFN2 degradation, activating mitochondrial fission and mitophagy.
Hepatocellular carcinoma	Mitochondria‐Lysosome	PINK1, Parkin, Rab7, p‐Drp1^Ser616^	p‐Drp1^Ser616^ anchors to mitochondria‐lysosome MSC via Rab7, enhancing PINK1/Parkin‐mediated mitophagy and reducing chemotherapy‐induced apoptosis.

## AUTHOR CONTRIBUTIONS

All authors contributed to drafting or revising the article, have agreed on the journal to which the article will be submitted, gave final approval of the version to be published, and agreed to be accountable for all aspects of the work.

## FUNDING INFORMATION

This work was supported by the National Natural Science Foundation of China (22177084 and 82273559), Sichuan Province Science and Technology Plan Project (2023NSFSC1546), Postdoc project of West China Hospital, Sichuan University, and China Postdoctoral Science Foundation.

## CONFLICT OF INTEREST STATEMENT

The authors declare no conflicts of interest.
